# The Overall Efficacy and Outcomes of Metronomic Tegafur-Uracil Chemotherapy on Locally Advanced Head and Neck Squamous Cell Carcinoma: A Real-World Cohort Experience

**DOI:** 10.3390/biology10020168

**Published:** 2021-02-23

**Authors:** Tsung-Jang Yeh, Leong-Perng Chan, Hui-Ting Tsai, Chin-Mu Hsu, Shih-Feng Cho, Mei-Ren Pan, Yi-Chang Liu, Chih-Jen Huang, Che-Wei Wu, Jeng-Shiun Du, Hui-Ching Wang

**Affiliations:** 1 Graduate Institute of Clinical Medicine, College of Medicine, Kaohsiung Medical University, Kaohsiung 807, Taiwan; aw7719@gmail.com (T.-J.Y.); pan.meiren.0324@gmail.com (M.-R.P.); 2 Division of Hematology and Oncology, Department of Internal Medicine, Kaohsiung Medical University Hospital, Kaohsiung Medical University, Kaohsiung 807, Taiwan; 940283@kmuh.org.tw (H.-T.T.); e12013@gmail.com (C.-M.H.); sifong96@gmail.com (S.-F.C.); ycliu@cc.kmu.edu.tw (Y.-C.L.); 3 Department of Otolaryngology-Head and Neck Surgery, Kaohsiung Medical University Hospital, Kaohsiung Medical University, Kaohsiung 807, Taiwan; oleon24@yahoo.com.tw (L.-P.C.); kmuent@yahoo.com.tw (C.-W.W.); 4 Department of Otorhinolaryngology-Head and Neck Surgery, Kaohsiung Municipal Ta-Tung Hospital, Kaohsiung 801, Taiwan; 5 Faculty of Medicine, College of Medicine, Kaohsiung Medical University, Kaohsiung 807, Taiwan; ccjjhh@cc.kmu.edu.tw; 6 Division of Specialist Nursing Office, Faculty of Internal Medicine, Kaohsiung Medical University Hospital, Kaohsiung 807, Taiwan; 7 Drug Development and Value Creation Research Center, Kaohsiung Medical University, Kaohsiung 807, Taiwan; 8 Department of Radiation Oncology, Kaohsiung Medical University Hospital, Kaohsiung 807, Taiwan; 9 Department of Radiation Oncology, Faculty of Medicine, College of Medicine, Kaohsiung Medical University, Kaohsiung 807, Taiwan

**Keywords:** HNSCC, metronomic chemotherapy, tegafur-uracil, survival

## Abstract

**Simple Summary:**

In head and neck squamous cell carcinoma (HNSCC), more than 60% of patients presenting with locally advanced disease carries a high risk of recurrence and distant metastasis, with a poor prognosis (five-year overall survival (OS), <50%). Therefore, further prevention of recurrence and distant metastasis is crucial for survival improvement in advanced HNSCC patients. In this retrospective study, we investigated the outcomes of metronomic chemotherapy with tegafur–uracil in locally advanced HNSCC (LA HNSCC). Our data showed that adding tegafur–uracil after curative surgery with adjuvant chemoradiotherapy or definitive concurrent chemoradiotherapy significantly improved OS, DFS, and DMFS in patients with LA HNSCC. As a metronomic maintenance regimen, tegafur–uracil was well tolerated with minimal adverse effects. We suggested tegafur–uracil as a maintenance therapy of choice for patients with LA HNSCC.

**Abstract:**

Metronomic chemotherapy inhibits tumor growth by continuous administration of lower-dose chemotherapy. Our study aimed to demonstrate the outcomes of metronomic chemotherapy with tegafur–uracil in locally advanced head and neck squamous cell carcinoma (LA HNSCC). This was a retrospective study including 240 patients with LA HNSCC. After standard treatment, 96 patients were further treated with metronomic tegafur-uracil, and 144 patients were not. No statistical differences were found between both groups with regard to sex, clinical stage, or primary treatment choice. There were more hypopharyngeal cancers and more patients with poor clinicopathological features, including lymphovascular invasion, extranodal extension, and positive margins in the tegafur–uracil group. The median follow-up duration was 31.16 months. Overall survival (OS) was not reached in the tegafur–uracil group and was 54.1 months in the control group (*p* = 0.008). The median disease-free survival (DFS) was 54.5 months in the tegafur–uracil group and 34.4 months in the control group (*p* = 0.03). Neither group reached distant metastasis-free survival (DMFS, *p* = 0.02). In patients with LA HNSCC, adding tegafur–uracil as metronomic chemotherapy after either curative surgery with adjuvant chemoradiotherapy or definitive concurrent chemoradiotherapy significantly improved the OS, DFS, and DMFS with tolerable adverse events.

## 1. Introduction

Head and neck squamous cell carcinoma (HNSCC) arising in the oral cavity, oropharynx, larynx, and hypopharynx was the seventh most common cancer worldwide in 2018 [1]. It accounted for about 3.9% of all new cancers and approximately 3.8% of all cancer deaths. In the United States, about 3% of all new cancers is head and neck cancer [2]. The incidence and mortality rate of HNSCC varies by geographical location, and HNSCC is the fourth most common cancer of men in Taiwan [3].

HNSCC treatment differs according to the disease stage, anatomical location, and surgical accessibility. Some early-stage disease (stages I or II) is curable with surgery or definitive radiotherapy, both providing similar tumor control and improving long-term survival rates in approximately 70 to 90% of patients [4]. However, more than 60% of patients present with locally advanced disease (stages III or IV) upon diagnosis [5]. The treatment of locally advanced disease varies according to the anatomical location and involves multidisciplinary care, including surgery, radiotherapy, and chemotherapy. Unfortunately, locally advanced disease carries a high risk of recurrence and distant metastasis, with a poor prognosis (five-year overall survival (OS), <50%) [5,6]. Therefore, further prevention of recurrence and distant metastasis is crucial for survival improvement in advanced HNSCC patients.

Metronomic chemotherapy is a maintenance therapy with continuous and dose-dense administration of chemotherapeutic drugs in lower doses (a tenth to a third of the maximum tolerated dose) [7,8]. Several mechanisms of action of metronomic therapy have been proposed, including inhibition of the nutrition supply for tumor growth, obstruction of tumor angiogenesis, immune system modulation, and cellular dormancy mechanisms [9]. It can directly affect tumor cells, tumor progenitors, and neighboring stromal cells. Additionally, it is believed to decrease metastasis [8,9,10,11]. The concept of metronomic chemotherapy has been applied to several malignant diseases, including breast cancer, lung cancer, prostate cancer, ovarian cancer, colorectal cancer, and nasopharyngeal carcinoma [8,12].

Tegafur–uracil (UFUR; TTY Biopharm, Taiwan), an active agent used as metronomic adjuvant chemotherapy, is a 4:1 molar mixture of uracil and tegafur [13]. Tegafur is a prodrug of fluorouracil, which is gradually converted to fluorouracil (5-FU) by the hepatic cytochrome P-450 enzymes. Uracil is a reversible inhibitor of the fluorouracil-degrading enzyme dihydropyrimidine dehydrogenase, the enzyme responsible for fluorouracil catabolism. Its administration can achieve a stable plasma 5-FU concentration with a low toxicity profile [14,15]. To date, tegafur–uracil has been widely used in several malignancies, including lung cancer, gastric cancer, colorectal cancer, breast cancer, and head and neck cancer. The antitumor effects of tegafur-–uracil as a metronomic agent in advanced oral cancer and nasopharyngeal carcinoma have been documented in previous studies [15,16,17].

In this study, we investigated the clinical efficacy and survival rates of using tegafur–uracil as metronomic chemotherapy in locally advanced HNSCC (LA HNSCC) patients after standard treatment.

## 2. Materials and Methods

### 2.1. Study Design and Patient Selection

This was an observational, retrospective, single-center, single-arm study. We reviewed chart records from 2012 to 2018 and collected clinical data with a diagnosis of LA HNSCC at the Kaohsiung Medical University Hospital, Taiwan. The data contained detailed information on clinical characteristics, histology, laboratory findings, and treatments. Inclusion criteria included locally advanced (stage III or non-distant metastatic stage IV) oral cavity, oropharynx, larynx, and hypopharynx squamous cell carcinoma. The TNM status and clinical stages were based on the American Joint Committee on Cancer TNM staging system. To evaluate the effect of metronomic chemotherapy using tegafur-uracil, we excluded operable patients without histological high-risk features, but included operable patients with high-risk histological features and inoperable patients. High-risk features were defined as positive surgical margins, extranodal extension (ENE), perineural invasion (PNI), or lymphovascular invasion (LVI).

### 2.2. Treatment

All patients received either curative surgery with adjuvant chemoradiotherapy (CRT) or definitive CRT as initial treatment. The CRT treatment included a total radiotherapy dose of 60–70 Gy and cisplatin-based chemotherapy. In the treatment group, oral tegafur–uracil was administered at a daily dose of 100–400 mg within three months after CRT. In the reference group, no further chemotherapy was applied. The study was conducted until April 2020.

### 2.3. Treatment Response and Safety Assessment

All patients were followed up regularly at the Outpatient Department (OPD) of the Medical Oncology and Department of Otorhinolaryngology. During the oral tegafur–uracil treatment period, the patients visited the OPD of these two departments monthly. The evaluation of disease status included tumor site inspection, laboratory examination, and imaging studies. Treatment response was assessed and determined by computed tomography or magnetic resonance imaging at baseline (before tegafur-uracil) and at three- to six-month intervals after treatment was started. Imaging studies within four weeks before tegafur–uracil were acceptable and were performed whenever clinical physicians suspected disease progression. The Response Evaluation Criteria in Solid Tumors (RECIST) guideline version 1.1 was used to determine disease progression and tumor response. After disease progression, further treatments and survival status were documented every three months. Regarding safety assessment, treatment-related adverse events were monitored monthly throughout the study and evaluated using the Common Terminology Criteria for Adverse Events version 4.0.

### 2.4. Statistical Analysis

The distribution of patient characteristics, comorbidities, reason of treatment failure, and adverse effects were summarized as the frequency and percentage for categorical variables and mean and standard deviation for continuous variables. The OS was defined as the time from diagnosis to death; the disease-free survival (DFS) was defined as the time from diagnosis to disease progression or death; and the distant metastasis-free survival (DMFS) was defined as the time from diagnosis to evidence of distant metastasis or death. The Kaplan–Meier survival analysis was used to calculate the cumulative DFS, DMFS, and OS rates, and the difference between each of the two survival curves was estimated using the log-rank test. The Cox proportional hazard regression analysis was used to estimate the hazard rate between two groups. Hazard ratios (HRs) and 95% confidence intervals (CIs) were computed. The adjusted HR was analyzed after adjustment for other clinical and pathological factors. The statistical significance level of the *p*-value was set at 0.05. All analyses were conducted using SAS software (version 9.4, SAS Institute, Cary, NC, USA).

## 3. Results

### 3.1. Baseline Characteristics of Patients

From 2012 to 2018, 240 patients were included. All patients were diagnosed with LA HNSCC (stage III or non-distant metastatic stage IV). Patients were either treated with curative surgery with adjuvant CRT or definitive concurrent CRT. After CRT, 96 patients were treated with metronomic chemotherapy of tegafur–uracil for three–12 months, and 144 patients were not.

Patient characteristics, including age, sex, tumor location, stage, pathologic grade, high-risk features, substance history, locoregional recurrence, and distant metastasis, are summarized in Table 1. The mean age was 56 years in the tegafur–uracil group and 54 years in the control group (*p* = 0.196). There were also no statistical differences between the tegafur–uracil and control groups with regard to sex, clinical cancer stage, and primary treatment choice. However, there were more hypopharyngeal cancers in the tegafur–uracil group and more oral cavity cancers in the control group. There were significantly more high-grade features, including LVI (*p* = 0.018), ENE (*p* < 0.001), and positive margin (*p* = 0.025) in the tegafur–uracil group. There were more patients who consumed alcohol in the tegafur–uracil group than in the control group (*p* = 0.024). Major medical comorbidities were also analyzed. Hypertension was the most common medical comorbidity in both groups, followed by diabetes, virus hepatitis B/C, and peptic ulcer disease. There were more patients with hypertension (*p* = 0.048) and cerebrovascular disease (*p* = 0.032) in the tegafur–uracil group, but other comorbidities showed no significant differences between the two groups.

### 3.2. Treatment Outcomes

**Figure 1:**
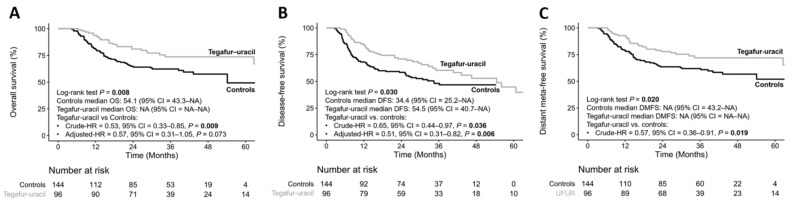
Kaplan–Meier plot for overall survival (**A**), disease-free survival (**B**), and distant metastasis-free survival (**C**).

The median follow-up duration was 31.16 (range: 3.80–87.38) months. Compared to the control group, the OS was not reached in the tegafur–uracil group and was 54.1 months in the control group (*p* = 0.008). In crude analysis, the HR of OS for tegafur–uracil was 0.53 (95% confidence interval (CI) = 0.33–0.85, *p* = 0.009) when compared with that in the control group. Post-estimation-adjusted OS analysis showed a non-statistically significant survival benefit in the tegafur–uracil group compared to the control group, with an HR of 0.57 (95% CI = 0.31–1.05, *p* = 0.073) (Figure 1A). For DFS, the median DFS was 54.5 months (95% CI = 40.7, not reached) in the tegafur–uracil group and 34.4 months (95% CI = 25.2, not reached) in the control group (*p* = 0.03). In the crude analysis, the analysis showed a statistically significant difference in the DFS benefit in the tegafur–uracil group than in the control group, with an HR of 0.65 (95% CI = 0.44–0.97, *p* = 0.036). After adjusted estimation, the DFS benefit was more apparent in the tegafur–uracil group than in the control group (adjusted HR = 0.51, 95% CI = 0.31–0.82, *p* = 0.006) (Figure 1B). The DMFS was not achieved in both groups (*p* = 0.02). In crude analysis, the HR of DMFS for tegafur–uracil was 0.57 (95% CI = 0.36–0.91, *p* = 0.019) when compared with that in the control group (Figure 1C), which demonstrated that maintenance with tegafur–uracil significantly reduced the risk of distant metastasis in HNSCC patients.

DFS and OS between the tegafur–uracil and control groups in different manners prior to chemotherapy are summarized in Supplementary Figure S1. The tegafur–uracil group showed a significantly longer median DFS in the subpopulation who received both surgery and CRT before chemotherapy, and also significantly better OS in the subpopulation who received both surgery and CRT or CRT alone before chemotherapy. Moreover, the treatment outcomes in different groups’ tumor stages are presented in Supplementary Figure S2. Compared to the controls, the tegafur–uracil group showed a favorable survival outcome, especially regarding the follow-up duration within 36 months in both OS and DFS in stage III and IV subgroups; however, only the OS results in stage IV subgroups reached a statistically significant outcome.

### 3.3. Durations of Tegafur–Uracil Administration Affected the Clinical Outcomes of HNSCC Patients

**Figure 2:**
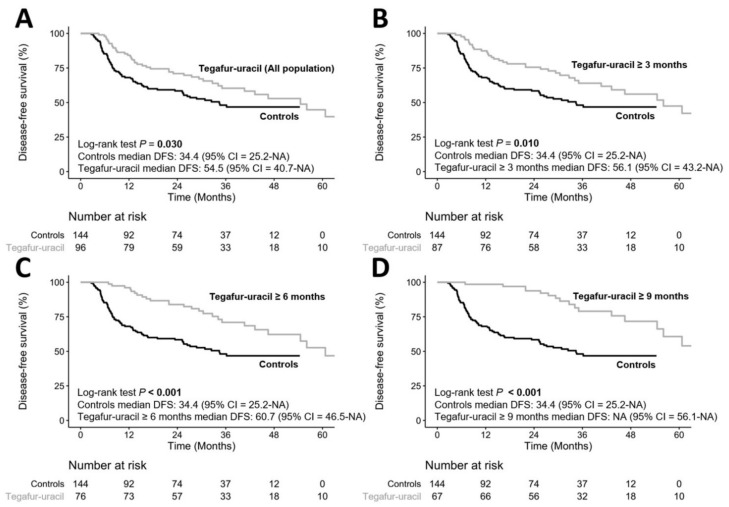
Comparison of tegafur–uracil duration on disease-free survival. (**A**) all population using tegafur–uracil vs controls, (**B**) tegafur–uracil ≥ 3months vs controls, (**C**) tegafur–uracil ≥ 6 months vs controls and (**D**) tegafur–uracil ≥ 9 months vs controls.

To clarify whether the duration of tegafur–uracil administration affected the clinical outcomes in HNSCC patients, we divided the patients into different durations of tegafur–uracil administration, including ≥three months, ≥six months, and ≥nine months of tegafur–uracil administration. The DFS rates of the ≥three-month, ≥six-month, and ≥nine-month durations were significantly longer than those in the control group (*p* = 0.010, *p* < 0.001, and *p* < 0.001, respectively), as shown in Figure 2. These results demonstrated that HNSCC patients using tegafur–uracil for a longer duration may gain more clinical benefits in the prevention of cancer relapse.

**Figure 3:**
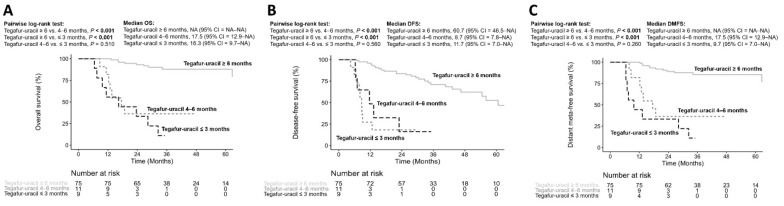
Analyses for the different duration of tegafur–uracil administration on overall survival (**A**), disease-free survival (**B**) and distant meta-free survival (**C**).

To analyze the optimal duration of tegafur–uracil administration, all patients were divided into ≤three, four–six months, and ≥six months. We performed pairwise comparisons using the log-rank test to analyze the OS, DFS, and DMFS. Patients with ≥six months of tegafur–uracil had better OS than both patients with three months and four–six months of tegafur–uracil (*p* < 0.001 and *p* < 0.001, respectively); however, patients with four–six months of tegafur–uracil showed no significant difference in OS compared with patients with three months of tegafur–uracil (*p* = 0.510), as shown in Figure 3A. Similar results were also found in DFS (Figure 3B) and DMFS (Figure 3C). The results similarly implied that a longer duration of tegafur–uracil administration (at least six months) provided more beneficial effects in LA HNSCC patients.

### 3.4. Safety and Analysis of the Treatment Failure

Treatment-related adverse effects are summarized in Table 2. Oral tegafur–uracil was well tolerated, with only grade 1 and grade 2 toxicities. The overall prevalence was 17.7%. The most common adverse effect was nausea/vomiting (3.8/3.3%), followed by neutropenia (2.9%) and mucositis (2.1%).

There were 110 patients who had treatment failure, including 68 with primary tumor recurrence, 19 with regional lymph node metastasis, and 23 with distant metastasis. There were no statistical differences in all categories between the tegafur–uracil and control groups (*p* = 0.669), as shown in Table 3.

### 3.5. Risk Factor Evaluation for Disease Progression

Risk factors for disease progression were analyzed using univariate regression. The parameters including age, risk behaviors (including alcohol, betel nuts, and tobacco consumption), comorbidity, tumor location, histologic features (including grade, LVI, PNI, and ENE), margin positivity, and previous treatment modality (including surgery and CRT) were all included. A subsequent multivariate regression analysis was performed to evaluate the significant factors affecting DFS in the univariate analysis.

As shown in Table 4, positive ENE was an independent factor related to shorter median DFS (HR = 1.62; *p* = 0.031, univariate analysis). This difference was significant following adjustment for other variables in the multivariate analysis (HR = 1.88; *p* = 0.009). Previous CRT was related to poor median DFS (HR = 2.18; *p* = 0.014, univariate analysis), and a more apparent difference was found in the multivariate analysis (HR = 2.46; *p* = 0.028). Significantly, maintenance therapy with tegafur–uracil was the favorite factor associated with a better median DFS (HR = 0.65, *p* = 0.036, and HR = 0.51, *p* = 0.006, during both univariate and multivariate analyses, respectively).

### 3.6. Determining the Risk Factor for Poorer Overall Survival

Similar factors were also analyzed to evaluate factors affecting OS. First, we observed that the grade of moderate differentiation had a significantly negative impact on OS compared with the grade of well differentiation (HR = 2.18, *p* = 0.004, and HR = 2.43, *p* = 0.019, in univariate and multivariate analyses, respectively). Patients with ENE demonstrated significantly poor OS (HR = 2.07, *p* = 0.006, and HR = 1.81, *p* = 0.047, in univariate and multivariate analyses, respectively). Maintenance therapy with tegafur–uracil showed a significantly longer OS in the univariate analysis (*p* = 0.009). After adjustment for other variables in the multivariate analysis, maintenance therapy with tegafur–uracil still demonstrated a trend toward better OS (HR = 0.57; *p* = 0.073). These results are shown in Table 5.

## 4. Discussion

In LA HNSCC, prevention of local recurrence and distant metastasis is crucial after the initial treatment. However, the five-year local control rate is approximately 50%, with up to 10–20% of patients still developing distant metastases [18,19]. High-risk factors for local recurrence, lymph node metastasis, and poor outcomes had already been demonstrated in previous studies, including positive surgical margins, extranodal extension, perineural invasion and lymphovascular invasion [20,21,22,23]. Unfortunately, most patients experience distant metastasis within the first two years during the follow-up period; up to 70% of patients have distant metastasis in the first year and 19% of patients in the second year. Patients without locoregional control have a significantly higher risk of distant metastasis than those with locoregional control [19]. Reducing the risk of distant metastasis to improve survival is an important issue in LA HNSCC.

The role of maintenance therapy in HNSCC remains uncertain. A phase III study compared the efficacy of S-1 and tegafur–uracil after curative therapy for stage III/IVA/IVB HNSCC. The DFS did not differ significantly between the groups, and the OS was significantly better in the S-1 group than in the tegafur–uracil group (3-year OS rate HR, 0.64; 95% CI, 0.44–0.94; *p* = 0.022) [24]. In unresected stage III/IVA/IVB HNSCC, concurrent CRT and lapatinib followed by lapatinib (1500 mg once daily) demonstrated improvement in CRR at six months post-CRT and median PFS in p16-negative disease, but no significant benefit in OS [25]. In India, maintenance with metronomic oral methotrexate and celecoxib in advanced oral cancer improved both DFS and OS [26,27,28]. However, the phase III randomized trial revealed that long-term lapatinib maintenance therapy provided no additional benefits in patients with surgically treated high-risk HNSCC [29]. Similarly, afatinib therapy for 18 months after definitive CRT in patients with intermediate- to high-risk unresected HNSCC did not improve DFS [30]. The phase III JAVELIN head and neck 100 trial (NCT02952586) showed that stage III/IVA/IVB HNSCC patients using avelumab plus CRT followed by avelumab maintenance failed to show a statistically significant improvement in PFS compared with CRT alone [31].

Tegafur–uracil and its metabolites, gamma-hydroxybutyric acid (GHB) and 5-FU, inhibited tumor proliferation and angiogenesis [32]. In 2000, tegafur–uracil exhibited their effect of preventing further distant metastasis via one-year administration after curative surgery in 424 non-metastatic Japanese HNSCC patients [33]. In a study in Taiwan, maintenance therapy with tegafur–uracil to CRT markedly improved DFS and OS rates in patients with locally advanced nasopharyngeal cancer [15]. In advanced oral cancer, maintenance therapy with metronomic tegafur–uracil significantly improved the five-year OS, DFS, and disease-specific survival rate, and the sequential distant metastasis rate was also decreased significantly [16,17]. Their results implied the potential antitumor efficacy of tegafur–uracil in HNSCC.

In this study, we analyzed all subgroups of patients with LA HNSCC, including the oral cavity, oropharynx, larynx, and hypopharynx. Maintenance tegafur–uracil treatment demonstrated better OS, DFS, and DMFS. Additionally, a longer duration of tegafur–uracil maintenance resulted in higher treatment efficacy in patients with LA HNSCC. However, there were still some limitations in our study, including a relatively small sample size and inevitable time bias. There were also some differences between the two groups in our study. The tumor locations were different in the two groups: More hypopharyngeal cancer patients in the tegafur–uracil group and more oral cancer patients in the control group. In addition, more aggressive phenotypes were observed in the tegafur–uracil group, including features of LVI, ENE, and positive margin. Patients with tegafur–uracil maintenance had worse clinicopathological features than the control group; however, better outcomes were observed in the tegafur–uracil group, which strengthened the therapeutic effect of tegafur–uracil in high-risk LA HNSCC patients.

## 5. Conclusions

As a metronomic maintenance regimen, adding tegafur–uracil after curative surgery with adjuvant CRT or definitive concurrent CRT significantly improved OS, DFS, and DMFS in patients with LA HNSCC. Tegafur–uracil was well tolerated with minimal adverse effects. We suggest tegafur–uracil as a maintenance therapy of choice for patients with LA HNSCC.

## Figures and Tables

**Table 1:** Baseline characteristics (*n* = 240).

	Total	Tegafur–Uracil	Controls	*p*
Cases, row %	240	96 (40%)	144 (60%)	
Age, years (mean ± SD)	55 ± 10	56 ± 10	54 ± 10	0.196
Sex, male	234 (97.5%)	94 (97.9%)	140 (97.2%)	0.036
Risk behavior				
Alcohol	172 (71.7%)	77 (80.2%)	95 (66.0%)	0.024
Betel	178 (74.2%)	76 (79.2%)	102 (70.8%)	0.196
Cigarette	210 (87.5%)	87 (90.6%)	123 (85.4%)	0.319
Comorbidity group (1)				0.499
No comorbidity	99 (41.2%)	35 (36.5%)	64 (44.4%)	
At least one or more	173 (72.1%)	72 (75.0%)	101(70.1%)	
Comorbidity group (2)				0.474
No comorbidity	99 (41.2%)	35 (36.5%)	64 (44.4%)	
1–3	117 (48.8%)	49 (51.0%)	68 (47.2%)	
>3	24 (10.0%)	12 (12.5%)	12 (8.3%)	
Comorbidities (details)				
Hypertension	93 (38.8%)	45 (46.9%)	48 (33.3%)	0.048
Diabetes mellitus	51 (21.2%)	21 (21.9%)	30 (20.8%)	0.974
Coronary heart disease	5 (2.1%)	2 (2.1%)	3 (2.1%)	1.000
Chronic kidney disease	12 (5%)	5 (5.2%)	7 (4.9%)	1.000
Chronic lung diseases (ex. COPD)	2 (0.8%)	1 (1.0%)	1 (0.7%)	1.000
Cerebrovascular disease	9 (3.8%)	7 (7.3%)	2 (1.4%)	0.032
Peptic ulcer disease/GERD	25 (10.4%)	7 (7.3%)	18 (12.5%)	0.281
Hepatitis B/C	40 (16.7%)	12 (12.5%)	28 (19.4%)	0.216
Gout	11 (4.6%)	5 (5.2%)	6 (4.2%)	0.950
Tumor location				0.001
Oral cavity	145 (60.4%)	46 (47.9%)	99 (68.8%)	
Oropharynx	45 (18.8%)	19 (19.8%)	26 (18.1%)	
Hypopharynx and larynx, others	50 (20.8%)	31 (32.3%)	19 (13.2%)	
Grade				0.078
Well-differentiated	73 (30.4%)	24 (25.0%)	49 (34.0%)	
Moderately differentiated	130 (54.2%)	52 (54.2%)	78 (54.2%)	
Poorly differentiated	34 (14.2%)	19 (19.8%)	15 (10.4%)	
Unknown	3	1	2	
Stage				0.350
III	51 (21.2%)	17 (17.7%)	34 (23.6%)	
IV	189 (78.8%)	79 (82.3%)	110 (76.4%)	
LVI	55 (22.9%)	29 (30.2%)	26 (18.1%)	0.018
PNI	60 (25.0%)	25 (26.0%)	35 (24.3%)	0.693
ENE	77 (32.1%)	46 (47.9%)	31 (21.5%)	<0.001
Margin positive	42 (17.5%)	23 (24.0%)	19 (13.2%)	0.025
Treatment before tegafur–uracil				
Surgery alone	38 (15.8%)	8 (8.3%)	30 (20.8%)	0.027
Surgery and CRT	151 (62.9%)	67 (69.8%)	84 (58.3%)	
CRT alone	45 (18.8%)	17 (17.7%)	28 (19.4%)	
Surgery	189 (78.8%)	75 (78.1%)	114(79.2%)	0.974
PF	55 (22.9%)	19 (19.8%)	36 (25.0%)	0.433
CRT	196 (81.7%)	84 (87.5%)	112 (77.8%)	0.082

*p*-value is estimated from *t*-test, chi-squared or Fisher’s exact test. Abbreviations: LVI, lymphovascular invasion; PNI, perineural invasion; ENE, extranodal extension; CRT, concurrent chemoradiotherapy; PF, cisplatin and fluorouracil.

**Table 2:** Adverse effects of tegafur–uracil (*n* = 96).

Adverse Events	Grade 1	Grade 2
Nausea	9 (3.8%)	
Vomiting	8 (3.3%)	
Mucositis	5 (2.1%)	
Neutropenia	4 (1.7%)	3 (1.2%)
Anemia	2 (0.8%)	1 (0.4%)
Thrombocytopenia	1 (0.4%)	
Chronic kidney disease, acute exacerbation	1 (0.4%)	
Diarrhea	3 (1.2%)	
Epigastralgia	1 (0.4%)	
Poor appetite	1 (0.4%)	
Skin itch	2 (0.8%)	
Skin rash	2 (0.8%)	

**Table 3:** Treatment failure (*n* = 240).

	Total	Tegafur–Uracil	Controls	*p*
Treatment failure				0.669
Primary tumor recurrence	68 (28.3%)	26 (27.1%)	42 (29.2%)	
Regional lymph nodes metastasis	19 (7.9%)	7 (7.3%)	12 (8.3%)	
Distant metastasis	23 (9.6%)	7 (7.3%)	16 (11.1%)	

**Table 4:** Cox regression analysis for disease-free survival.

Variable	Comparison	Univariate		Multivariate	
		Crude-HR (95% CI)	*p*	Adjusted-HR (95% CI)	*p*
Group	Tegafur–uracil vs. controls	0.65 (0.44–0.97)	0.036	0.51 (0.31–0.82)	0.006
Age	≥ 55 vs. < 55 years	0.63 (0.43–0.92)	0.016	0.82 (0.52–1.29)	0.393
Risk behavior	Yes vs. no	1.69 (0.74–3.86)	0.200	-	
Alcohol	Yes vs. no	1.31 (0.85–2.03)	0.200	-	
Betel	Yes vs. no	2.33 (1.37–3.96)	0.002	1.87 (0.94–3.71)	0.075
Cigarette	Yes vs. no	2.05 (1.00–4.22)	0.051	-	
Comorbidity status (1)	Yes vs. no	0.82 (0.55–1.23)	0.300	-	
Comorbidity status (2)	1–3 vs. 0	0.80 (0.52–1.21)	0.300	-	
	> 3 vs. 0	1.002 (0.52–1.94)	0.994	-	
Tumor location	Oropharynx vs. oral cavity	0.92 (0.57–1.50)	0.700	-	
	Others vs. oral cavity	0.68 (0.41–1.14)	0.150	-	
Grade	Moderately vs. well	1.46 (0.96–2.23)	0.075	-	
	Poorly vs. well	0.62 (0.30–1.30)	0.200	-	
LVI	Yes vs. no	1.42 (0.89–2.26)	0.140	-	
PNI	Yes vs. no	1.77 (1.13–2.76)	0.012	1.35 (0.85–2.15)	0.204
ENE	Yes vs. no	1.62 (1.05–2.51)	0.031	1.88 (1.17–3.01)	0.009
Margin positivity	Yes vs. no	1.09 (0.66–1.79)	0.700	-	
Treatment before tegafur–uracil	Surgery alone vs. none	0.30 (0.08–1.14)	0.078	-	
	Surgery and CRT vs. none	0.77 (0.24–2.45)	0.700	-	
	CRT alone vs. none	0.99 (0.30–3.28)	0.984	-	
Surgery	Yes vs. no	0.68 (0.44–1.04)	0.076	-	
CRT	Yes vs. no	2.18 (1.17–4.07)	0.014	2.46 (1.10–5.51)	0.028

Abbreviations: LVI, lymphovascular invasion; PNI, perineural invasion; ENE, extranodal extension; CRT, concurrent chemoradiotherapy; HR, hazard ratio; 95% CI, 95% confidence intervals. Multivariate model includes only significant variables in univariate analysis.

**Table 5:** Cox regression analysis for overall survival.

Variable	Comparison	Univariate		Multivariate	
		Crude-HR (95% CI)	*p*	Adjusted-HR (95% CI)	*p*
Group	Tegafur–uracil vs. controls	0.53 (0.33–0.85)	0.009	0.57 (0.31–1.05)	0.073
Age	≥ 55 vs. < 55 years	0.78 (0.51–1.22)	0.300	-	
Risk behavior	Yes vs. no	2.40 (0.76–7.59)	0.140	-	
Alcohol	Yes vs. no	1.69 (0.98–2.93)	0.060		
Betel	Yes vs. no	2.30 (1.22–4.35)	0.010	2.14 (0.90–5.09)	0.084
Cigarette	Yes vs. no	2.30 (0.93–5.69)	0.072	-	
Comorbidity status (1)	Yes vs. no	1.10 (0.67–1.81)	0.700	-	
Comorbidity status (2)	1–3 vs. 0	1.02 (0.61–1.71)	0.928	-	
	>3 vs. 0	1.61 (0.78–3.34)	0.200	-	
Tumor location	Oropharynx vs. oral cavity	0.98 (0.56–1.72)	0.943	-	
	Others vs. oral cavity	0.75 (0.41–1.35)	0.300	-	
Grade	Moderately vs. well	2.18 (1.28–3.72)	0.004	2.43 (1.16–5.10)	0.019
	Poorly vs. well	0.65 (0.24–1.74)	0.400	0.90 (0.27–3.04)	0.866
LVI	Yes vs. no	1.89 (1.11–3.22)	0.019	1.24 (0.67–2.30)	0.486
PNI	Yes vs. no	2.29 (1.35–3.87)	0.002	1.51 (0.84–2.72)	0.164
ENE	Yes vs. no	2.07 (1.23–3.50)	0.006	1.81 (1.01–3.23)	0.047
Margin positivity	Yes vs. no	0.91 (0.49–1.69)	0.800	-	
Treatment before tegafur–uracil	Surgery alone vs. none	0.38 (0.08–1.91)	0.200	-	
	Surgery and CRT vs. none	0.85 (0.21–3.49)	0.800	-	
	CRT alone vs. none	1.19 (0.28–5.11)	0.800	-	
Surgery	Yes vs. no	0.64 (0.39–1.05)	0.080	-	
CRT	Yes vs. no	2.03 (0.98–4.22)	0.058	-	

Abbreviations: LVI, lymphovascular invasion; PNI, perineural invasion; ENE, extranodal extension; CRT, concurrent chemoradiotherapy; HR, hazard ratio; 95% CI, 95% confidence intervals. Multivariate model includes only significant variables in univariate analysis.

## Data Availability

The datasets presented in this article are not readily available because of patient confidentiality and participant privacy terms. Requests to access the datasets should be directed to the corresponding author.

## Supplementary Materials

The following are available online at https://www.mdpi.com/2079-7737/10/2/168/s1. Figure S1: Kaplan–Meier plot for overall survival and disease-free survival in different manner prior to chemotherapy. Figure S2: Kaplan–Meier plot for overall survival and disease-free survival in different tumor stage.
