# Supplementary figures and images for: The Overall Efficacy and Outcomes of Metronomic Tegafur-Uracil Chemotherapy on Locally Advanced Head and Neck Squamous Cell Carcinoma: A Real-World Cohort Experience
Source: Biology (Basel). 2021 Feb 23;10(2):168. doi: 10.3390/biology10020168 (PMC7926982; doi:10.3390/biology10020168)

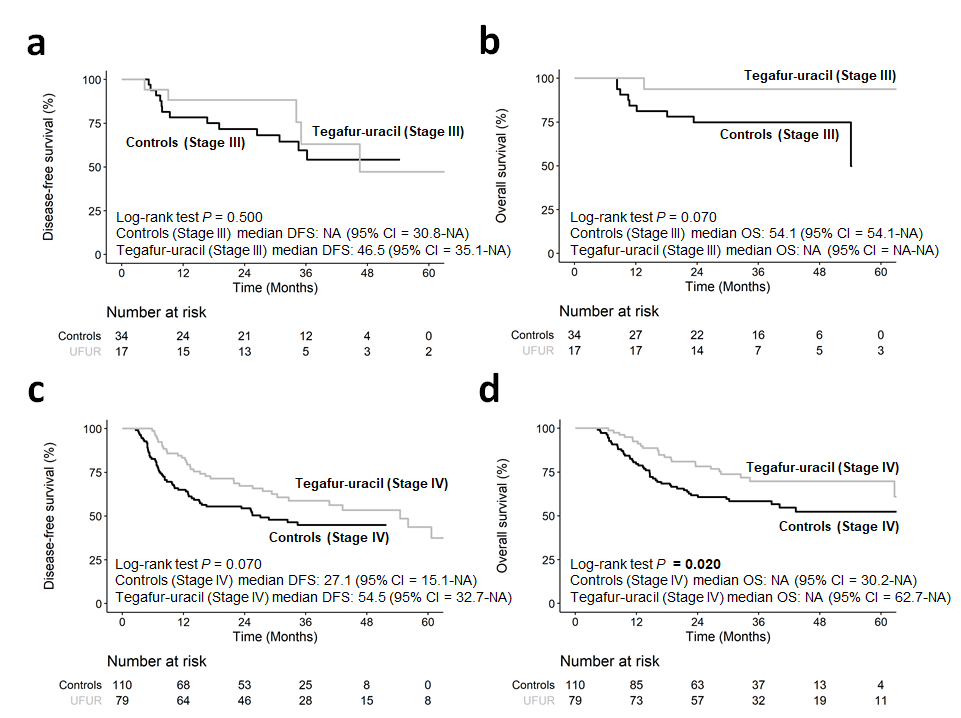

Supplement: Supplementary file 1 [file biology-10-00168-s001.zip › biology-1099683-suppl-final/20210209-FigureS1.tif]

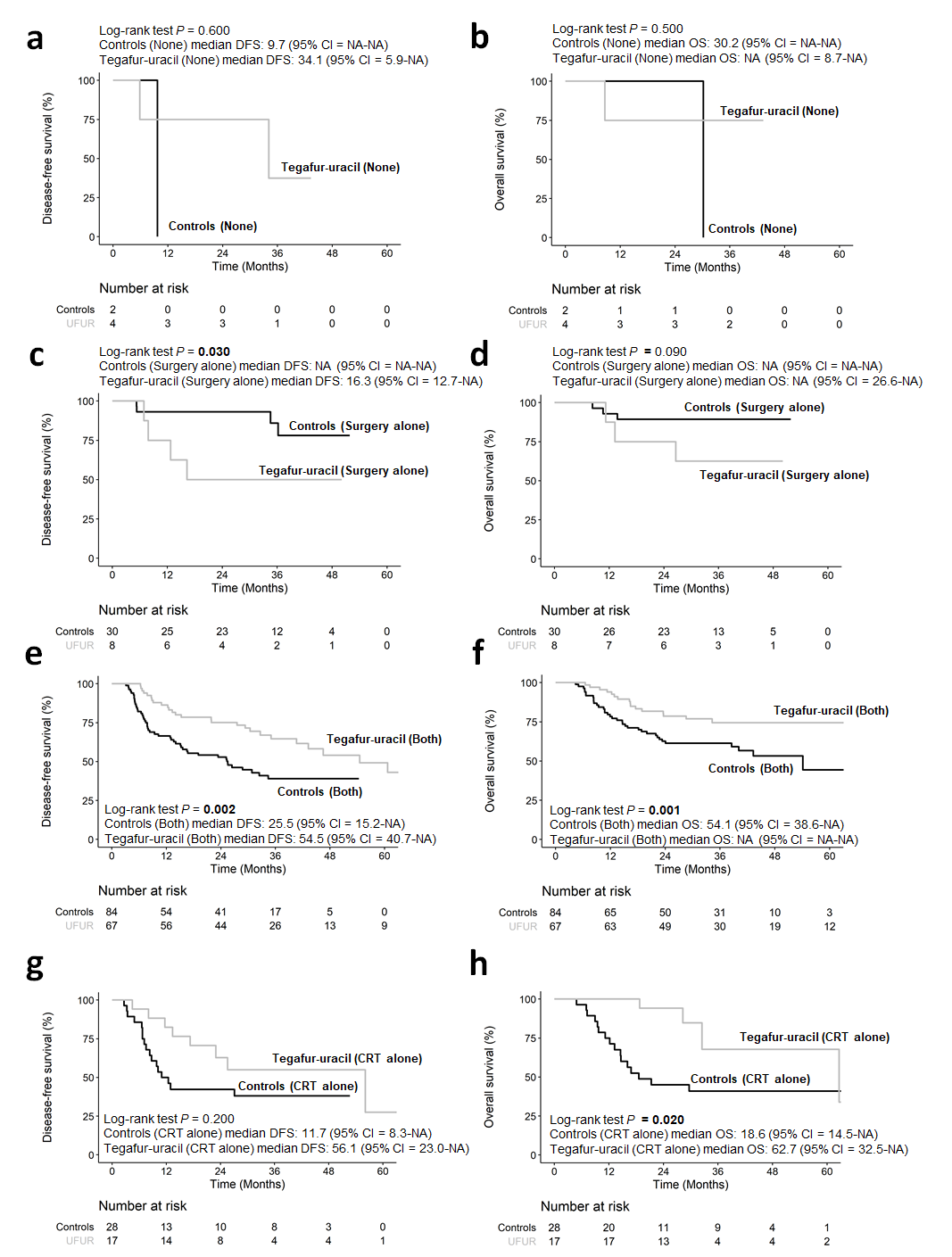

Supplement: Supplementary file 1 [file biology-10-00168-s001.zip › biology-1099683-suppl-final/20210209-FigureS2.tif]
